# Assessment of the Condition of Wharf Timber Sheet Wall Material by Means of Selected Non-Destructive Methods

**DOI:** 10.3390/ma12091532

**Published:** 2019-05-10

**Authors:** Tomasz Nowak, Anna Karolak, Maciej Sobótka, Marek Wyjadłowski

**Affiliations:** Wroclaw University of Science and Technology, Wybrzeze Wyspianskiego 27, 50-370 Wroclaw, Poland; tomasz.nowak@pwr.edu.pl (T.N.); maciej.sobotka@pwr.edu.pl (M.S.); marek.wyjadlowski@pwr.edu.pl (M.W.)

**Keywords:** timber structures, non-destructive methods, ultrasonic wave, stress wave, drilling resistance, X-ray micro-computed tomography

## Abstract

This paper presents an assessment of the condition of wood coming from a wharf timber sheet wall after 70 years of service in a (sea) water environment. Samples taken from the structure’s different zones, i.e., the zone impacted by waves and characterised by variable water-air conditions, the zone immersed in water and the zone embedded in the ground, were subjected to non-destructive or semi-destructive tests. Also, the basic parameters of the material, such as its density and moisture content, were determined. Moreover, the ultrasonic, stress wave and drilling resistance methods were used. Then, an X-ray microtomographic analysis was carried out. The results provided information about the structure of the material on the micro and macroscale, and the condition of the material was assessed on their basis. Also, correlations between the particular parameters were determined. Moreover, the methods themselves were evaluated with regard to their usefulness for the in situ testing of timber and to estimate, on this basis, the mechanical parameters needed for the static load analysis of the whole structure.

## 1. Introduction

Timber as a universal building material has been used in many kinds of structures in, e.g., geotechnical engineering. In this field, one of the principal uses of structural timber is as timber pile foundations in low bearing capacity building lands situated in river deltas and beds and on peat bogs, which often were important locations because of their strategic position. As a foundation method, timber piles have been used almost everywhere in Europe in, i.a., the Scandinavian countries, the countries of Eastern and Middle Europe, such as Poland, the Baltic countries and Russia, as well as Germany, Great Britain and the Netherlands, where the foundation of historical buildings is most fully documented. Also, in the south of Europe, for instance in Venice, deep foundation timber piles may be found in nearly all the historical buildings erected since the 12th century [1].

Wood is a heterogeneous, hygroscopic, cellular and anisotropic material. Its mechanical properties depend on many factors, such as density and water content, which means that the creation of a constitutive model of wood poses a great challenge [2].

Wood biodegrades over time. Under the impact of external factors, wooden members undergo chemical and physical changes. Wood can be regarded as a durable material when it is completely immersed in water, and so protected against decomposition caused by aerobic fungi.

In nature, five basic chemical processes occur which reconvert a wooden material into carbon dioxide and water: oxidation, hydrolysis, dehydration, reduction and free radical reactions [3].

Table 1 shows major degradation pathways and the chemistries involved in the pathways [3].

All the above factors can be significant when testing wood samples taken from a retaining structure since the latter is exposed to the air, water and soil environment. Therefore, wood analyses should be based on more than one research technique to gain a deeper insight into the change of wood parameters over time in different environmental conditions [3].

In the considered case, since the structure was in service in the water environment and in saturated soils, the hazards can be divided into the following three main groups:

- a low or variable water level,
- excessive loading,
- decomposition of wood in the water environment.

If timber members are above the groundwater table, access to oxygen makes the activation of wood decomposing fungi possible. The decomposition rate is determined by the time during which the timber members are above groundwaters and the member’s length situated above the groundwater table [4]. It is estimated that the maximum rate of decomposition caused by fungi attacking water-saturated wood amounts to approximately 10 mm/year [1]. However, the long-lasting service of timber structures below the groundwater table does not prevent decomposition [5], and examinations have shown some of the pile foundations in Venice to be in extremely bad condition.

This paper presents research methods which enable one to determine the condition and some parameters of a material which has been in service in the water-soil environment. For the best results, wood should be tested on different levels of detail, i.e., macro, submacro and microlevels. On the macrolevel, acoustic methods, drilling resistance method or laboratory tests of basic material parameters, such as density and moisture content, are used. On the microlevel the cell wall of the wood is tested and different elements, such as hardwood, sapwood and annual rings, are identified [6], using, e.g., X-ray micro-computed tomography.

The considered timber sheet wall was made of tongue-and-groove jointed timber piles. The history of this structure is not well known because of its previous military use. The timber sheet wall had been in service for about 70 years. The wharf is in the Swina straight connecting the Szczecin Lagoon with the Baltic Sea. In the Swina strait, fresh water (fully or partially) mixes with seawater due to the stratification. The salinity of the Swina strait ranges from 1‰ to 8‰. The average salinity of the Baltic Sea amounts to about 7‰, generally ranging from 2 to 12‰. It can be assumed that the water environmental conditions correspond to low salinity seawater.

**Figure 1:**
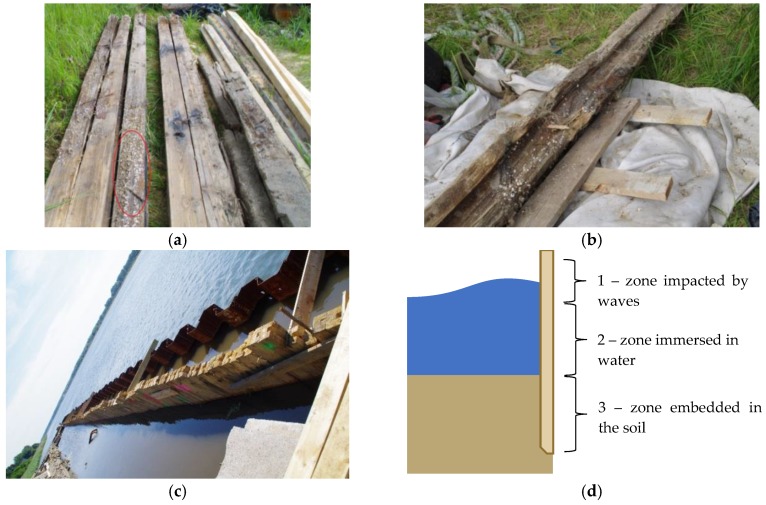
(**a**,**b**) View of wharf timber sheet pile wall from which samples were taken for testing (the zone impacted by waves is marked with red ellipse); (**c**) view of rebuilt wharf timber sheet pile wall; (**d**) scheme of the global structure with the location of the specimens.

After the timber sheet wall had been dug out and dismantled, its members were closely examined with regard to their original and current cross-sectional dimensions and to the quality of the wood. In the photograph (Figure 1a,b) of the dismantled members of the wall, one can see pile surfaces which were in service in diverse environmental conditions: completely embedded in the ground, stayed in water and stayed in the variable water-air environment. One can notice that the timber embedded in the ground, under the groundwater table, has preserved its constant volume. Bacteria destroy the cellulose very slowly, while the lignin remains constant, and water replaces the large cellulose molecules. The original waterfront layout has been reconstructed, see Figure 1c.

The main objective of the work is to develop a methodology for testing of wooden structural members using non-destructive techniques. This is aimed at obtaining information which is necessary to assess the technical condition of the material in wooden members and to conduct a global structure analysis. In particular, to carry out such analysis, it is required to estimate the values of mechanical parameters and to assess possible zones of destruction. The detailed aim is to compare the quality of wood subjected to various environmental conditions (Figure 1d).

## 2. Selected Non-destructive Methods for Wood Assessment

### 2.1. Brief Survey of Methods

Material tests for wood can be divided into three groups: destructive tests (DT), semi-destructive tests (SDT) [7] and non-destructive/quasi-non-destructive tests (NDT) [8]. Unlike destructive tests, the tests belonging to the latter two groups do not affect or only slightly affect the properties of the tested sample, whereby the parameters of a wooden member can be determined with no detriment to its value. Their undeniable advantage is also the mobility of the testing equipment, whereby tests can be carried out in situ when it is not possible to take samples for laboratory tests (as in the case of heritage assets). Among the non-destructive and semi-destructive methods one can distinguish global testing methods (e.g., ultrasonic and stress wave techniques) and local testing methods (e.g., the drilling resistance method).

In order to acquire detailed data on the values of the physical and mechanical parameters of wood both non-destructive and destructive methods should be used. If the results yielded by the two testing methods are found to correlate, the data acquired in this way are fully sufficient for further static load analyses of the structural members or the whole building structure. Nevertheless, even using only non-destructive methods (as in the case of, e.g., heritage assets), one can obtain some information about the properties of the tested member’s material, assess the technical condition of the structure or acquire some data helpful in evaluating this condition or in the design of possible repairs or upgrades. Thanks to the use of non-destructive methods one can also detect internal damage or flaws in the wood [9].

Among the non-destructive and quasi-non-destructive testing methods used to assess and diagnose timber structures, the most common are the ones presented in Table 2, and also described in detail in, i.a., [7,8,10,11]. The non-destructive and quasi-non-destructive methods can be divided into two groups: global testing methods (e.g., visual evaluation and ultrasonic and stress wave techniques) and local testing methods (e.g., the drill resistance method, the core drilling method and the hardness test method).

### 2.2. Acoustic Methods

#### 2.2.1. Idea of Acoustic Test

Using acoustic testing methods, such as the ultrasonic and stress wave techniques, one can evaluate the properties of wood by analysing the velocity of wave propagation in the tested material. The methods can be used to estimate selected mechanical properties (e.g., the modulus of elasticity) of a material and to detect its internal structural discontinuities.

The basic parameter used in the acoustic methods is sound wave propagation velocity (V), defined as follows:

V = L/T,(1)

where L is the distance (between two measuring points) covered by the excited sound wave, and T is the time needed to cover this distance.

Knowing the velocity of wave propagation and the wood density (ρ), one can determine the dynamic modulus of elasticity (MOE_dyn_), which can be interrelated with the static modulus of elasticity (MOE_stat_) [10]. The dynamic modulus of elasticity is calculated from the formula:

MOE_dyn_ = V^2^ × ρ, (2)

where Vis the velocity of sound wave propagation, and ρ is the density of the tested element.

The velocity of sound wave propagation largely depends on the structure of the material. In the case of wood, it depends on the grain direction being several times higher (usually 3–5 times higher) along than across the grain [10,12].

According to [12], for wood with no significant structural flaws the velocity of sound wave propagation amounts to 3500–5000 m/s along the grain and to 1000–1500 m/s across the grain. Other values than the above ones may indicate internal discontinuities in the material structure. The lower values of the velocity across the grain are due to the internal structure of this material (on its way the wave encounters more cell walls, whereby the time in which it covers the distance increases, whereas in the longitudinal direction there are fewer barriers or they do not occur, whereby the velocity is higher).

#### 2.2.2. Description of Testing Methods and Devices

Several kinds of devices are used for testing by means of ultrasonic or stress wave methods. In this case study, two of them were used and the test results are presented in Section 3.

**Figure 2:**
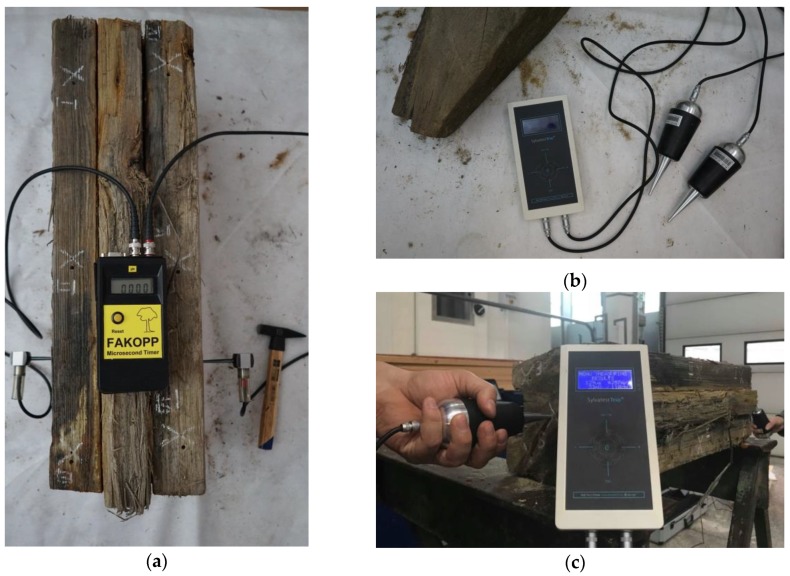
Devices for testing by acoustic method: (**a**) Fakopp Microsecond Timer using stress wave; (**b**) Sylvatest Trio device using ultrasonic wave; (**c**) Sylvatest Trio device during test.

The Fakopp Microsecond Timer (Figure 2a) uses the stress wave technique. The test consists of exciting a stress wave with a single strike of a special hammer. The device probes are driven directly into the tested sample. There is no need to drill holes as in the case of other devices (e.g., Sylvatest Trio). The device measures the time of wave propagation between the two probes (the receiving probe and the transmitting probe).

In the case of the ultrasonic technique, the measurement can be performed in two ways: directly and indirectly. The first way consists of transmitting a signal from the transmitting probe to the receiving probe, with the probes placed on the opposite sides of the tested sample. As regard the second way, there is no need to place probes on the opposite sides of the sample because the signal is registered as reflected (the echo method). Owing to this, the range of the applicability of this test widens since only a unilateral access is required (which is useful when testing, e.g., historical monuments in situ).

Sylvatest Trio (Figure 2b), manufactured by the firm CBS-CBT, is another device which one can use to non-destructively evaluate the properties of wood [13]. The device measures the time needed for an ultrasonic wave to pass between transmitting-receiving probes placed against the tested sample, and the energy of this wave. In order to carry out the test the tips of the probes should be inserted into previously drilled holes each 5 mm in diameter and 10 mm deep. One should bear in mind that because of the high sensitivity of the device other mechanical waves excited near the test site can affect the test results. Also, the material’s moisture content and internal stresses can significantly influence the results.

In order to obtain exhaustive results, it is recommended, for both methods, to perform a large number of measurements in different points and directions.

#### 2.2.3. Correlation between Physical and Mechanical Properties of Wood and Results Yielded by Acoustic Methods

In many studies (e.g., [14,15,16,17,18,19,20]) based on acoustic methods attempts were made to assess the effectiveness of the methods and to find a correlation between the physical and mechanical properties of wood and the parameter values obtained from measurements.

According to the above studies, there is a strong correlation between the dynamic modulus of elasticity (MOE_dyn_) and the static modulus of elasticity (MOE_stat_). According to [18], for sound wood free of flaws the determination coefficient for the static and dynamic modulus of elasticity amounts to 0.96.

Also, comparative analyses of the effectiveness of the Fakopp Microsecond Timer (Fakopp Enterprise Bt., Agfalva, Hungary) and the Sylvatest Trio device (-CBS-CBT, Choisy-le-Roi, France) were carried out. They showed the two devices to be highly effective [18,19] and confirmed the correlation between the value of MOE_dyn_ and that of MOE_stat_ [20].

As part of other investigations, the decrease in the value of the velocity of the ultrasonic wave and the stress wave was analysed. According to [12], a reduction in the velocity by about 30% can correspond to a 50% fall in the load bearing capacity, while a reduction in the level of velocity by more than 50% can indicate considerable damage and the loss of load bearing capacity by the tested element. According to the results of the above research the relative decrease in the value of the velocity of wave propagation between two measuring points (ΔV_rel_) describes the degree of damage to the material. The value of ΔV_rel_ is defined by Equation (3):

ΔV_rel_ = [(V_ref_ − V_mes_)/V_ref_]·100%, (3)

where ΔV_rel_ is the relative decrease in velocity, V_ref_ is the reference velocity (value of the velocity for a sound wood, taken from tests or literature), and V_mes_ is the measured velocity.

The relation between the relative decrease in velocity and the degree of damage is shown in Table 3 [21].

### 2.3. Drilling Resistance Method

#### 2.3.1. Description of Test

One of the semi-destructive (SDT) methods is the drilling resistance method.

After the test a small borehole, below 3.0 mm in diameter, (not larger than the exit hole of most of the woodworm) remains in the sample material, but with no detriment to the properties of the element, whereby the test can be regarded as semi-destructive [7].

**Figure 3:**
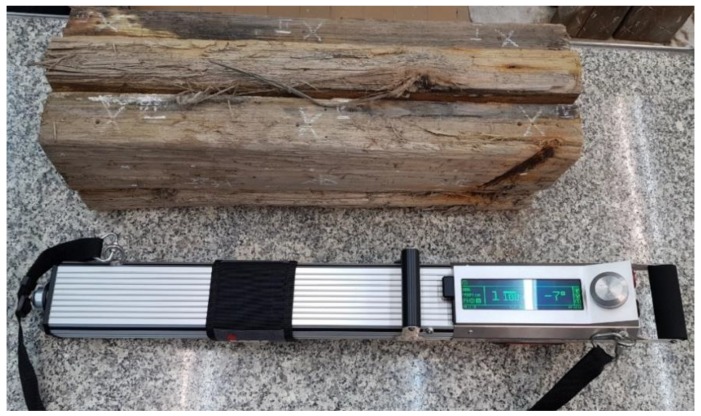
IML RESI PD-400S device used in tests.

The test consists of measuring the energy needed to drill the resistance drilling device’s metal needle into the material. The test makes it possible to detect structural discontinuities, damage, knots and other flaws and also to estimate the density and strength of the material [22]. The device measures the drilling resistance of a drill with a diameter of 1.5–3.0 mm and a length of 300–500 mm, rotating at a constant speed of about 1500 rpm (Figure 3).

Drilling resistance is closely connected with the difference in density between the zones of early and late wood [22], the structure of the annual rings [23,24], changes in wood density caused by, i.a., biological decomposition, and the drilling angle [25]. The device registers the measurement results at every 0.1 mm, in the form of drilling resistance-depth graphs. The peaks in the graph correspond to the high resistance and high density of the material while the declines represent its low resistance and low density. The flatline in the diagram indicates places where the material does not show any drilling resistance, which means that the material is completely decomposed. During drilling the measurement in the entry and exit zones is disturbed because of the time needed for the drill to assume the proper position and rotational speed. Consequently, the graph in these zones usually has the form of a smoothly rising or declining curve.

Using the device one can detect structural flaws and discontinuities in timber elements without adversely affecting their useful properties (see, e.g., [14,26,27]).

#### 2.3.2. Correlation between Physical and Mechanical Properties of Wood and Drilling Resistance Results

Attempts have been made to correlate drilling resistance results with strength test results in order to estimate the mechanical parameters of wood in the structure (e.g., [27,28,29,30,31,32,33]). Diagrams of relative resistance (RA) versus drilling depth (H) make it possible to evaluate the parameters of wood through the correlation between the average value of the resistance measure (RM) parameter and the density, strength and the modulus of elasticity of the wood. The value of RM can be calculated from formula 4 [25]:

(4) $$\mathrm{RM}\;=\;\frac{\int_{0}^{H}\mathrm{RA}\cdot \mathrm{dh}}{H},$$

where $\int_{0}^{H}\mathrm{RA}\cdot \mathrm{dh}$ is the area under the drilling resistance graph, and H is the drilling depth.

Attempts have also been made to correlate the resistance measure with different material parameters (density, longitudinal modulus of elasticity, transverse modulus of elasticity, longitudinal compressive strength, transverse compressive strength and bending strength) for different wood species, new wood and old wood. The results of some of the endeavours presented high determination coefficients amounting to 0.78 for the transverse compressive strength and to 0.67 for the modulus of elasticity [16] as well as to 0.64 for the modulus of elasticity and the longitudinal compressive strength [25]. As regards density, the determination coefficients of 0.71 [25], 0.75 for Pine, 0.74 for Spruce, 0.65 for Fir [28], 0.70 [29], 0.80 [30] or even 0.88 [31] were obtained. However, some researchers [32,34,35] did not obtain such a good correlation.

In general, tests performed on non-decayed, defect free, small sized laboratory specimens provide high values of correlation coefficients. On the other hand, results of the onsite tests of full-sized elements must be analysed with greater caution due to the possible presence of defects. In this context the drilling resistance method should be perceived to be a qualitative method. 

The RM parameter value is influenced by many factors, such as the tree species, the condition of the wood and its moisture content and the drilling direction [32]. The results should be treated as not a quantitative, but qualitative assessment and the resistance drilling method test can be a complement to other tests or the starting point for a preliminary inspection of timber members or the location of damage inside the cross section.

### 2.4. X-Ray Micro-Computed Tomography

**Figure 4:**
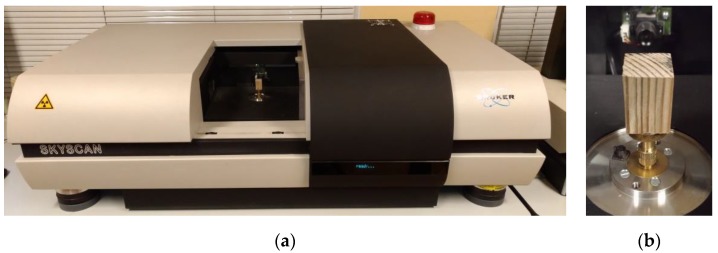
(**a**) Bruker SkyScan 1172 device; (**b**) sample of timber mounted on stage inside scanning chamber.

X-ray micro-computed tomography (Skyscan 1172, Bruker, Kontich, Belgium) is a state-of-the-art non-destructive technique for visualizing the inner structure of the tested object [36,37]. In essence, the tests consists of mathematically reconstructing the three-dimensional microstructure of the tested material on the basis of a series of high-resolution X-ray pictures. The scanning consists of recording a series of projections taken during the slow rotation of the sample placed on the scanner’s rotary fixture [38]. The Bruker SkyScan 1172 microtomograph used in the tests and a view of a sample placed in the scanning chamber are shown in Figure 4 below.

A single projection taken at a set sample rotation angle shows (in greyscale) the distribution (registered by the detector) of the intensity of the X-ray radiation emitted by the source and attenuated by passing through the sample. After a series of projections is recorded, the mathematical reconstruction of the tested material is carried out. The fact that according to the Lambert-Beer law, radiation absorption depends on the material’s attenuation coefficient and on the thickness of the layer which the radiation must penetrate, is exploited for this purpose. The most commonly used algorithms are based on back projection, e.g., the Feldkamp algorithm [39] used in the present study. The result of the reconstruction is a series of images representing the cross sections of the examined object. The images show (in greyscale) the distributions of the attenuation coefficient, i.e., a characteristic of the material. The cross sections, arranged one above the other in space, make up a three-dimensional image of the internal microstructure of the examined object. Using image analysis techniques, quantitative and qualitative analyses of the material's microstructure can be carried out on the basis of the reconstructed images [40,41].

Since the absorption coefficient depends mainly on density, the greyscale of the resulting image can be treated as a monotonic density function. Consequently, the correlation between the level of brightness of the pixels (voxels) and the density of the tested material can be determined. For example, in [41,42] the linear correlation between density and the brightness level of the resultant tomographic image was used to determine the density of wood.

When testing heterogenous materials (composites), a morphometric analysis is carried out [38,43]. Its aim is to quantitatively characterise the morphology of a given component of the composite, particularly by determining the shape parameters and form of the geometrical objects constituting the area (in space) occupied by the considered component. Such an analysis is carried out on binary images obtained from segmentation.

## 3. Materials and Methods

In this case study, non-destructive and semi-destructive testing methods, i.e. the ultrasonic and stress wave techniques and the drilling resistance technique, were used to estimate the parameters of samples taken from the structure. Also c.a. 20 mm × 20 mm × 30 mm samples were cut out from the same structural members and subjected to scanning in the microtomograph in order to augment the non-destructive test results. The direct result of the scanning is a 3D image of the microstructure of the tested material. The level of brightness in the images is approximately proportional to the local density of the tested material. Owing to this a semi-quantitative comparison of wood density for the different zones of the timber sheet wall could be made.

**Figure 5:**
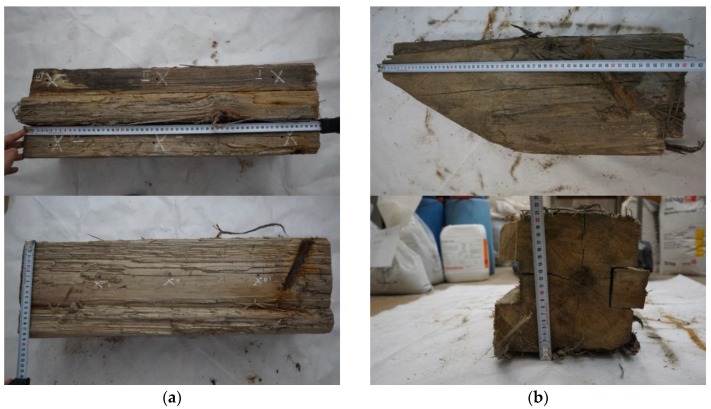
Samples and their dimensions: (**a**) sample 1 cross section 18 cm × 20 cm and length 60 cm, (**b**) sample 3 cross-section 18 cm × 20 cm and length 60 cm with cut.

Using the testing methods and devices described in Section 2, a series of tests were carried out on samples taken from the wharf timber sheet wall. The samples dimensions were about 18 cm × 20 cm cross-section and 60 cm length (Figure 5). The samples come from different locations in the wharf structure, which means that in the course of their service they were submerged to different levels and exposed to the variable impact of water. The material of the samples is pinewood (*Pinus sylvestris L*). The tests were carried out in a laboratory at Wroclaw University of Science and Technology.

The acoustic tests and the stress wave tests were carried out using respectively the Sylvatest Trio device and the Fakopp Microsecond Timer.

A new generation device IML RESI PD400 with a drill length of about 400 mm was used for drilling resistance testing. The device can register both drilling resistance and the feed force at every 0.1 mm. Five-millimetre deep entry and exit zones were assumed when calculating mean drilling resistance RM from formula (5). The zones were not taken into account in the calculations. The places where drilling resistance (RA) amounted to less than 5% and where the under-five-per-cent values in the diagram extended for minimum 5 mm were regarded as zones with flaws.

Moreover, the moisture content in the samples was determined using an FMW moisture meter (of the resistance type with a hammer probe).

Density was determined using 20 mm × 20 mm × 400 mm flawless samples prepared from the tested timber samples (16, 12 and 12 samples from each of the member, altogether 40 samples) with a moisture content of 18%. In accordance with the standard procedure [44], density was calculated from formula (5):

ρ = ρ(u)·[1 − 0.005 (u − u_ref_)], (5)

where ρ is density, u is the sample’s moisture content during testing, and u_ref_ is the reference moisture content = 12%.

Also, the correction due to the size of the sample is needed; the value should be divided by 1.05 [44]. 

Density determinations and the drilling resistance tests were carried out for the wood moisture content of about 18%. Acoustic tests were carried out three times for chosen different sample moisture content levels: about 30% (direct after taking samples from the structure), 24–28% and 17–18% to examine the effect of moisture content on the measurement results.

Also, a series of scans of the small samples were performed using the Bruker SkyScan 1172 microtomograph.

**Figure 6:**
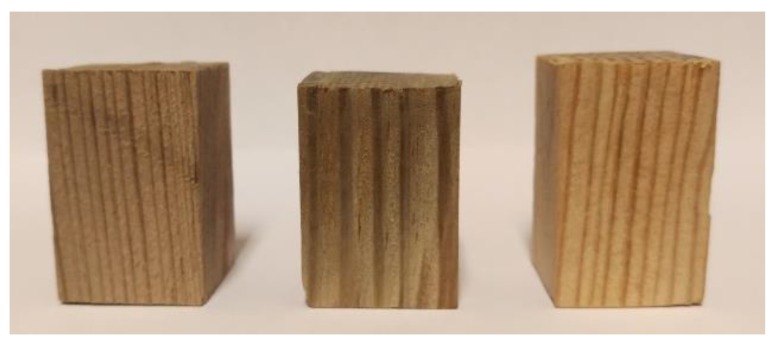
Samples prepared for scanning: “1”, “2” and “3” (from left to right).

The total number of three samples were scanned using X-ray micro-computed tomography (see Figure 6 below):

- “1” a portion of timber from the zone impacted by waves—a sample with c.a. 20 mm × 20 mm × 30 mm dimensions,
- “2” a portion of timber from the zone submerged in water)—a sample with c.a., 20 mm × 20 mm × 30 mm dimensions,
- “3” a portion of timber from the zone sunk in the ground—a sample with c.a., 20 mm × 20 mm × 30 mm dimensions.

The samples were scanned in the Bruker SkyScan 1172 device (Figure 4a). The same set of scanning parameters was used for each of the samples in order to ensure identical scanning conditions. The selected major scanning parameters are summarised in Table 4 below.

## 4. Results and Discussion

### 4.1. Density and Moisture Content

Density was determined for the 40 flawless small samples and the results are presented in Table 5.

On the basis of the measurements performed by means of a resistance-type moisture meter the moisture content in the samples was determined to amount to 18 ± 1%. In addition, testing by acoustic methods was carried out for two more different moisture content levels, i.e., about 30% and 24–28%.

### 4.2. Drilling Resistance

**Figure 7:**
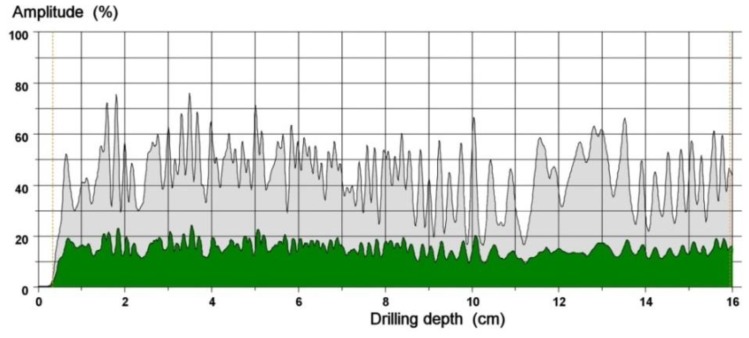
Exemplary drilling resistance curve (green) and feed force curve (grey).

Drilling resistance tests were carried out on 3 samples with a moisture content of 18 ± 1%. Twenty measurements were performed on each of the samples. An exemplary drilling resistance curve and a feed force curve are shown in Figure 7. The depth to which the biological corrosion of the wood extends (3 mm in this case) can be easily read off the diagram. The drilling resistance test results for the particular samples are presented in Table 6 and Table 7.

Only 60 measurements were carried out as part of the laboratory tests, but the number of samples in in situ tests is usually not larger because of the not fully non-destructive character of the testing method. Since the test has a pointwise character and wood is a heterogenous material, it is necessary to perform numerous measurements to assess its condition and density. Therefore, one cannot responsibly evaluate wood on the basis of single measurements.

Many factors have a bearing on drilling resistance, e.g., moisture content, drill sharpness, drilling angle and direction and battery charge status [45]. Moreover, wood flaws, such as knots (resulting in very high drilling resistance) and damaged zones (zero or close to zero drilling resistance) affect the RM value, which was taken into account in the analysis. The places with knots were neglected in drilling resistance and feed force calculations.

**Figure 8:**
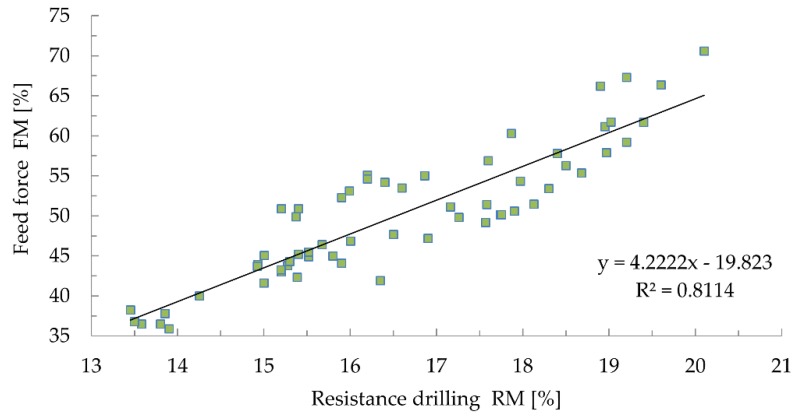
Correlation between drilling resistance and feed force.

**Figure 9:**
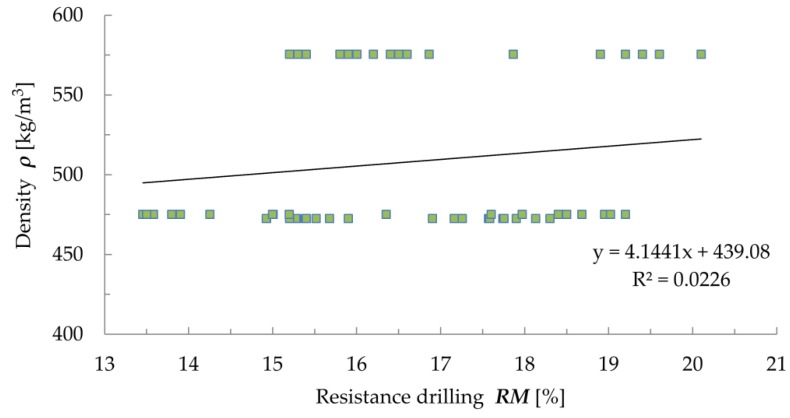
Correlation between drilling resistance and density.

**Figure 10:**
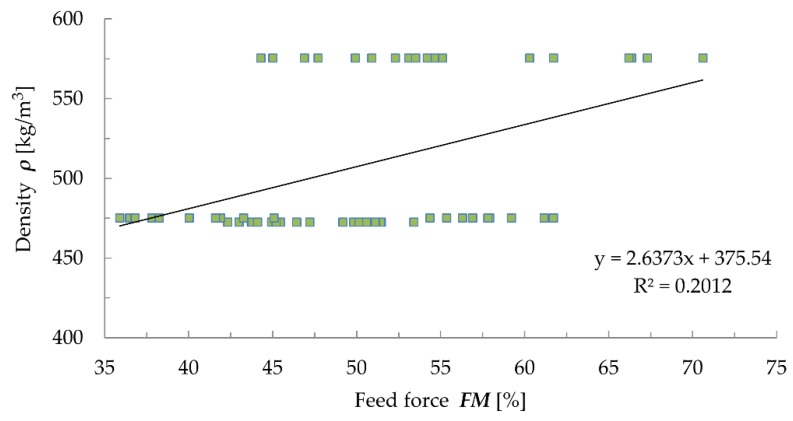
Correlation between feed force and density.

Despite the quite good correlation (Figure 8) between drilling resistance and feed force (R^2^ = 0.8114), no correlation between these quantities and density was found (Figure 9 and Figure 10), which casts doubt on the correlativity between them [32,46]. The results obtained using the drilling resistance method can be used to estimate the depth of wood damage in static load analyses to reduce the cross sections of the members.

### 4.3. Stress and Ultrasonic Waves

**Figure 11:**
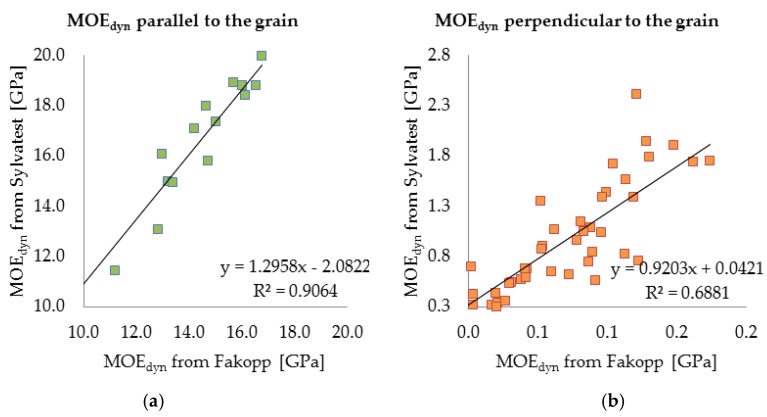
Correlation between dynamic modulus of elasticity determined using ultrasonic wave method (Sylvatest) and stress wave method (Fakopp) for all the samples for all moisture content levels for both directions relative to grain: (**a**) parallel; (**b**) perpendicular.

The propagation times of the stress wave and the ultrasonic wave and the length of the distance covered by the waves in both the directions (along and across) relative to the grain were registered and used to calculate the velocity of wave propagation in the material. Then the dynamic moduli of elasticity were calculated using the densities measured for the particular samples (sample 1—472.5 kg/m^3^, sample 2—475.2 kg/m^3^, sample 3—575.5 kg/m^3^). The results are shown in Table 8 and Table 9. Also the dynamic elasticity modulus values parallel and perpendicular to the grain, yielded by the two methods were correlated for selected samples. The results are presented in Figure 11.

As one can see above, the results yielded by the two measuring methods using the Sylvatest Trio device and the Fakopp Microsecond Timer are similar. For the selected samples the correlation coefficient along and across the grain amounts to respectively R^2^ = 0.9064 and R^2^ = 0.6881. This result can be regarded as satisfactory and it indicates the two methods can be used complementarily to estimate the material parameters of wood.

### 4.4. X-Ray Micro-Computed Tomography

3D images of the samples were reconstructed on the basis of a series of X-ray projections, using the Feldkamp algorithm in the NRecon software (version 1.7.1.0) by Bruker. Selected major reconstruction parameters are summarised in Table 10 below.

**Figure 12:**
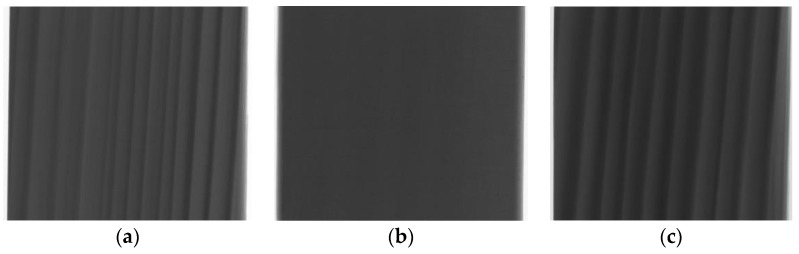
Exemplary projections for samples: (**a**) “1”; (**b**) “2”; (**c**) “3” (scale 200%).

Exemplary projections for all of the tested samples are shown in Figure 12.

**Figure 13:**
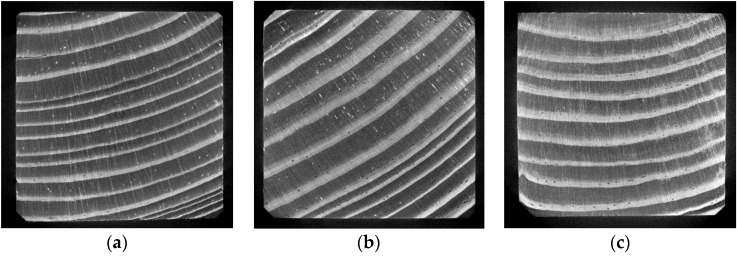
Exemplary cross sections of samples: (**a**) “1”; (**b**) “2”; (**c**) “3” (scale 200%).

**Figure 14:**
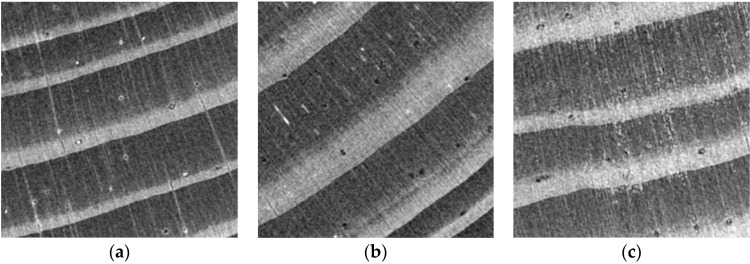
Exemplary cross section (scale 800%): (**a**) sample “1”; (**b**) sample “3”; (**c**) sample “2”.

Exemplary cross sections of the samples, obtained by reconstructing the 3D sample model are shown in Figure 13 and Figure 14, at a scale of respectively 200% and 800% (an enlarged fragment of the image).

**Figure 15:**
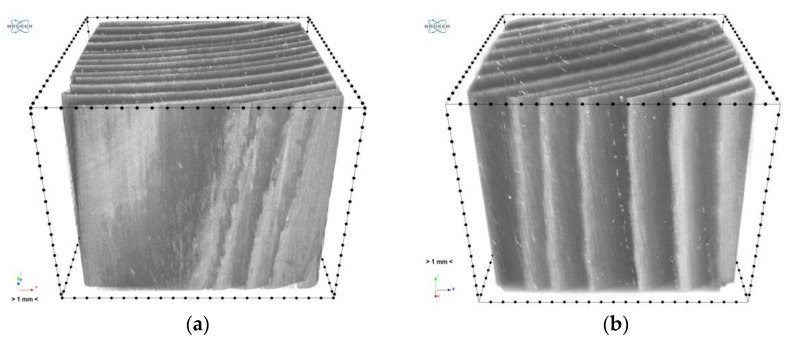

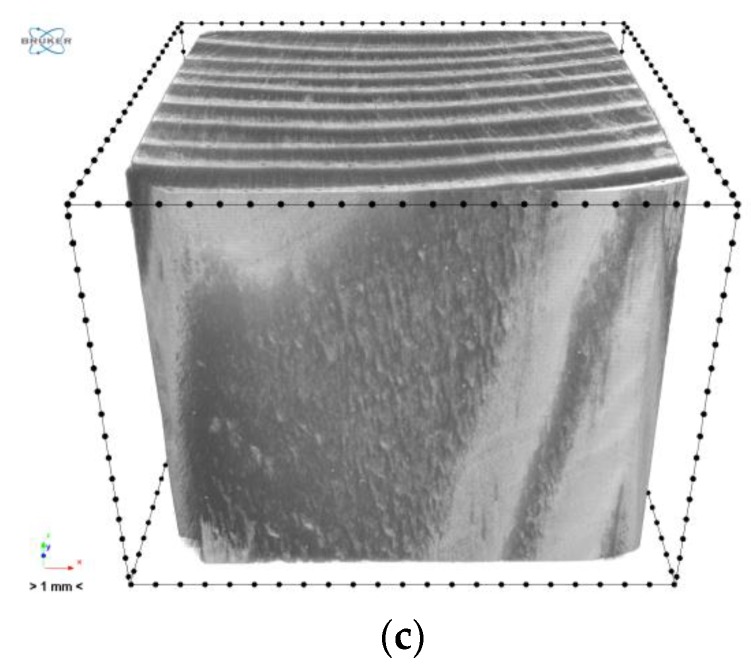
Reconstruction—3D view: (**a**) sample “1”, (**b**) sample “2”, (**c**) sample “3”.

Figure 15 shows the rendering of the 3D model of the samples.

The cell structure is practically invisible due to the adopted scanning resolution. Only the early and late wood with local flaws (small microcrack in sample “1”) and higher-density inclusions (samples “1” and “2”) can be distinguished. In the images obtained from scanning, sample “3” is generally brighter than the other two, which unambiguously indicates its higher density. This is particularly visible in the cross sections (Figure 13 and Figure 14). It also appears from the reconstruction that the late growth rings, which are denser (brighter in the imaging results), are thicker and occupy more material volume in samples “2” and “3”, whereas in sample “1” the volume fraction of late growth rings is clearly smaller. Moreover, small highly dense (probably mineral) inclusions are noticeable in samples “1” and “2”.

**Figure 16:**
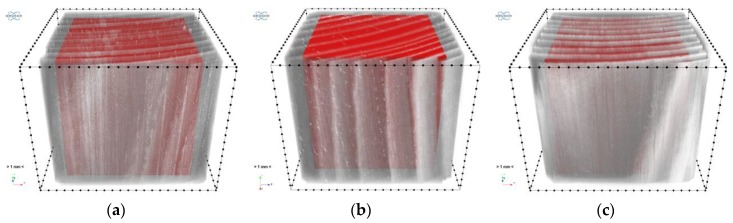
Selection of VOI: (**a**) sample “1”; (**b**) sample “2”; (**c**) sample “3”.

A cubic area of (1200 vox)^3^ completely contained within the volume of the tested material, i.e., the so-called volume of interest (VOI), was selected in order to quantitatively characterise the above observations. The selection of VOI is shown in Figure 16 below.

**Figure 17:**
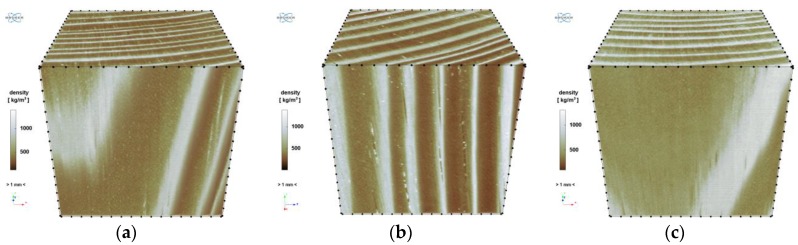
Spatial distribution of density: (**a**) “1”; (**b**) “2” and (**c**) “3”.

**Figure 18:**
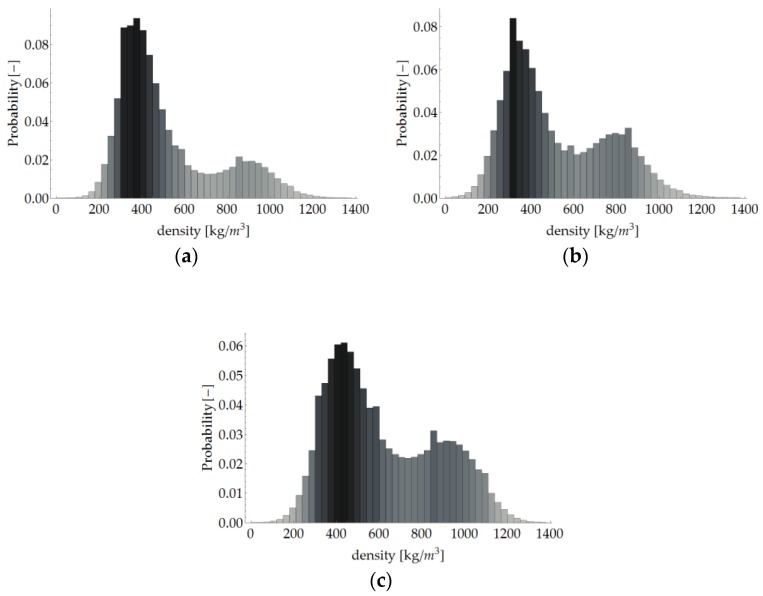
Histogram of local density in VOI: (**a**) sample “1”; (**b**) “2” and (**c**) “3”.

As mentioned, there is a correlation between greyscale and density. Thanks to the use of such a correlation in the linear form as in [41,42], the spatial distributions of local density in the analysed samples (Figure 17) and the statistical distributions (histograms) of density in the VOI of the particular samples (Figure 18) were determined. The correlation coefficients were determined by comparing the mean density of a given sample with the average grey level in VOI, and the density of the air with the average grey level of area outside the sample (visible in the images obtained from X-ray micro-computed tomography). The coefficient of proportionality of this correlation, determined independently for each of the three samples, amounted to respectively 5.884, 5.808 and 5.906. The distributions presented below were obtained using the mean value of this coefficient, i.e., 5.866.

**Figure 19:**
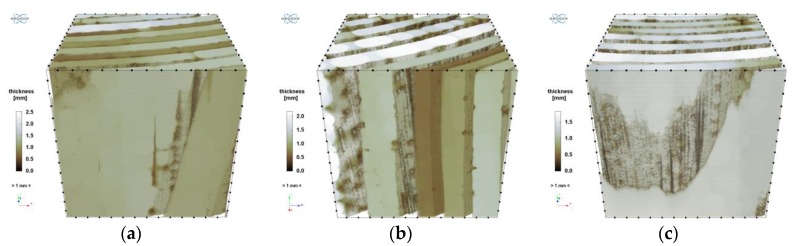
Distribution of local thickness of early growth rings: (**a**) sample “1”; (**b**) sample “2”; (**c**) sample “3”.

**Figure 20:**
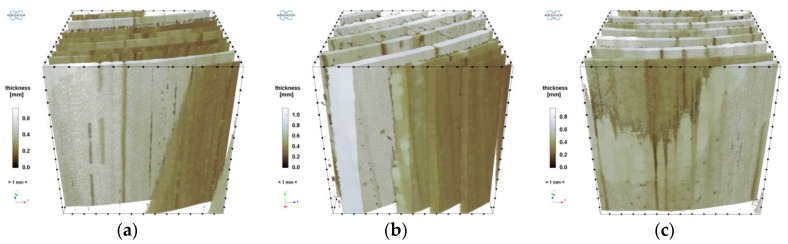
Distribution of local thickness of later growth rings: (**a**) sample “1”; (**b**) sample “2”; (**c**) sample “3”.

As one can see, the above histograms represent bimodal probability distributions. This means that there occur two principal components. In the case of this analysis, these are the early and late growth rings. Segmentation, i.e. partitioning the image into segments occupied by the particular components, was performed using the thresholding preceded by a smoothing filter. The threshold value of brightness corresponds to the grey level value at which the image histogram reaches a minimum (sample “1”: 135, “2”: 122, “3”: 149). A morphometric analysis was carried out for the two image components. In particular, the volume fraction of the component and the spatial distribution of its local thickness [47] were determined and then its mean thickness was calculated. The calculated values are shown in Table 11 and the thickness distributions are presented in Figure 19 and Figure 20. In order to avoid the error connected with determining thickness at the boundary of VOI, the area of the latter was limited to the volume of (1000 vox)^3^ by applying dilation with a radius of 100 voxels.

### 4.5. Comparative Analysis for Samples from Different Zones

Results obtained for samples from different zones: the zone impacted by waves (sample 1), the zone immersed in water (sample 2) and the zone embedded in the ground (sample 3) are summarised in the Table 12.

The obtained results confirm the engineering “intuition” about the impact of the environment on the degradation level of the material. The best condition of wood was observed for sample embedded in the soil (sample 3). The highest values of density were obtained for this sample. The same applies to the modulus of elasticity and the FM parameter, which are positively correlated with density. At the same time differences between parameters for samples from the zone impacted by waves (sample 1) and the zone immersed in water (sample 2) are not significant. Moreover, in the whole member there were no changes observed in the material within the surface layer exposed to direct environmental impact in the contact zone. Eventually, despite the noticeable differences between the different zones, it can be stated that in the tested timber members, after 70 years of operation, no significant destruction, reducing the safety of use, was found. In that sense, the condition of a wharf timber sheet wall’s material may be described as fairly good.

## 5. Conclusions

For a reliable assessment of the technical condition of timber structures, the use of non-destructive examinations is recommended, in addition to visual evaluation. Still, there are no comprehensive studies in this area, which present correlations enabling estimation of the mechanical parameters of wood and the degree of destruction, although some attempts of predicting these parameters or instance by regression analysis were made (among others [48]).

None of the currently known non-destructive methods used to assess the condition of timber members does not allow for an unambiguous estimation of the strength characteristics of wood. This is not possible even when using the X-ray method [41], which enables relatively accurate measurement of wood density. It results from the material inhomogeneous internal structure, including different defects, f. ex. knots, which have a significant impact on the strength parameters of wood.

In the case of the resistance drilling tests, the obtained coefficients of determination between drilling resistance and density (R^2^ = 0.0226) and between the feed force and density (R^2^ = 0.2012) do not indicate any correlation between the results. Drilling resistance testing should be treated not as a quantitative, but qualitative assessment. The results obtained by means of this method can be used to estimate the depth of material damage in static load analyses to reduce the cross sections of the analysed members [14,49].

The acoustic testing (using the Sylvatest Trio device and the Fakopp Microsecond Timer) provided data on the value of the dynamic modulus of elasticity which can be correlated with the static modulus of elasticity. The latter is the basic mechanical parameter needed to carry out a global structural analysis. The obtained coefficients of determination between the values of MOE_dyn_ yielded by the two measuring methods (R^2^ = 0.9064 along the grain and R^2^ = 0.6881 across the grain) for the selected samples are satisfactory, showing the results to be reliable. The velocity of acoustic wave propagation (and so the modulus of elasticity) clearly decreases as the moisture content in the wood increases. The acoustic testing methods can be regarded as useful for estimating material stiffness parameters (Young’s modulus), but they require further research in order to develop correlations comprising wood moisture content. Currently, research is underway to correlate the dynamic modulus of elasticity with the static modulus of elasticity. On the basis of the determined value of modulus of elasticity, it is possible to estimate mechanical parameters of material, for example according to standard procedure [50].

One should bear in mind that the two methods supply information about the local state of the material. In order to determine the global parameters, one should perform the largest possible number of measurements, which is not always possible, especially in the case of in situ testing. Therefore, in order to obtain the most accurate data on the tested member or structure, it is recommended to combine several testing methods.

Despite their non-destructive character, in most cases the testing methods require samples to be taken to determine the density of the material.

Thanks to the use of X-ray micro-computed tomography, the internal microstructure of the wood could be imaged. The results of the laboratory density measurements were used as input data for determining the correlation between the greyscale of the tomography results and the local density of the wood. Consequently, it became possible, for example, to estimate the density of the early and late wood. It should be noted that the correlation was determined independently for each of the three tested samples and very good agreement was obtained. The values of the coefficient of proportionality do not differ by more than 2%. This means that such a correlation can be a tool for the precise evaluation of the local density of the tested material on the microscale. As a result of the morphomorphic analysis based on the scanning results the volume fractions and morphology of the particular wood components, i.e. the early and late growth rings, could be determined. The data acquired from the analysis in the microtomograph can be useful in micromechanical modelling aimed at estimating the effective parameters of the material on the basis of microscale information.

Summing up, resistance drilling tests enable to determine the depth of material decayed zones, acoustic methods provide estimation of mechanical parameters. The application of the X-ray microtomography allows detailed insight to be gained into the microstructure of the material in a different scale of observation. In particular, it makes it possible to determine the occurrence of microdefects and to determine the parameters (density) of wood constituents, i.e. early and late growths. It must be pointed out that the applied methods are not equivalent, but rather, they are complementary. 

The paper presents the methodology for comprehensive wood testing in structural members using the described research methods. The set of results obtained from these methods makes it possible to assess the material, and consequently, to perform a global analysis of the structure. In particular, it is possible to estimate the value of mechanical parameters, whereas the qualitative evaluation makes it possible to determine the extent of possible material destruction and the location of any defects.

## Figures and Tables

**Table 1:** Major degradation pathways and chemistries.

**Biological Degradation**	Fungi, bacteria, insects, termites, enzymatic reactions, oxidation, hydrolysis, reduction, free radical reactions
**Chemical Reactions**	Oxidation, hydrolysis, reduction, free radical reactions
**Mechanical Degradation**	Chewing dust, wind, hail, snow, sand, stress, crack, fracture, abrasion, compression
**Thermal Degradation**	Lightning, fire, sun
**Water Degradation**	Rain, sea, ice, acid rain, dew
**Water Interactions**	Swelling, shrinking, freezing, cracking, erosion
**Weather Degradation**	Ultraviolet radiation, water, heat

**Table 2:** Selected methods available for assessing timber in building structure.

Organoleptic Methods	Acoustic Methods	Quasi-Non-destructive (Semi-Destructive) Methods	Radiographic Methods	Other Methods
Visual evaluation Acoustic evaluation Fragrance evaluation	Stress waves Ultrasonic technique Acoustic emission	Drilling resistance Core drilling Screw withdrawal Hardness tests Needle penetration Pin pushing Tension microspecimens	X-rays Gamma rays	Computed tomography Ground penetrating radar Near infrared spectrometry

**Table 3:** Relation between relative velocity decrease and degree of damage.

Relative Velocity Decrease [%]	Degree of Damage [%]
0–10	no destruction
10–20	10
20–30	20
30–40	30
40–50	40
≥50	≥50

**Table 4:** Scanning parameters.

Parameter	Value
Source Voltage	59 kV
Source Current	167 µA
Projection image size	2000 × 1333 pix
Image Pixel Size	13,56 µm
Filter	Al foil
Exposure	750 ms
Rotation Step	0.24°
Frame Averaging	ON (6)
Random Movement	ON (10)
Use 360 Rotation	YES

**Table 5:** Determined densities.

Sample	Number of Measurements	Density ρ				
		Mean Value from Tests [kg/m^3^]	Mean value Calculated according to [44] [kg/m^3^]	Range [kg/m^3^]	Standard Deviation [kg/m^3^]	Coefficient of Variation [%]
1	16	511.5	472.5	449.6–547.0	27.2	5.3
2	12	514.4	475.2	484.4–549.1	25.8	5.0
3	12	623.0	575.5	598.8–671.1	20.5	3.3
summary	40	545.8	504.2	449.6–671.1	58.5	10.8

**Table 6:** Mean drilling resistance tests results.

Sample	Number of Measurements	Drilling Resistance *RM* [%]			
		Mean	Range	Standard Deviation	Coefficient of Variation
1	20	16.5	14.9–18.3	1.2	7.4
2	20	16.3	13.5–19.2	2.3	14.1
3	20	16.9	15.2–20.1	1.6	9.5
summary	60	16.6	13.5–20.1	1.8	10.6

**Table 7:** Mean feed force test results.

Sample	Number of Measurements	Feed Force *FM* [%]			
		Mean	Range	Standard Deviation	Coefficient of Variation
1	20	47.4	42.3–53.4	3.5	7.3
2	20	47.7	35.9–61.7	9.8	20.6
3	20	55.3	44.3–70.6	7.7	13.9
summary	60	50.1	35.9–70.6	8.2	16.4

**Table 8:** Fakopp Microsecond Timer test results: velocity of stress wave propagation and dynamic modulus of elasticity depending on moisture content and direction relative to grain.

Sample	Direction Relative to Grain	V [m/s]			MOE_dyn_ [GPa]		
		Moisture Content			Moisture Content		
		~30%	24–28%	~18%	~30%	24–28%	~18%
1	parallel	4872.8	5376.6	5644.8	11.22	13.66	15.06
	perpendicular	1153.7	1439.9	1443.8	0.63	0.98	0.98
2	parallel	-	-	-	-	-	-
	perpendicular	1218.5	1372.8	1270.6	0.71	0.90	0.77
3	parallel	-	-	-	-	-	-
	perpendicular	1406.1	1447.7	1665.2	1.14	1.21	1.60

**Table 9:** Sylvatest Trio test results: velocity of ultrasonic wave propagation and dynamic modulus of elasticity depending on moisture content and direction relative to grain.

Sample	Direction Relative to Grain	V [m/s]			MOE_dyn_ [GPa]		
		Moisture Content			Moisture Content		
		~30%	24–28%	~18%	~30%	24–28%	~18%
1	parallel	5128.9	5855.4	6035.2	12.43	16.20	17.21
	perpendicular	1118.4	1311.5	1356.9	0.59	0.81	1.04
2	parallel	-	-	-	-	-	-
	perpendicular	1030.2	1311.4	1481.1	0.50	0.82	1.04
3	parallel	-	-	-	-	-	-
	perpendicular	1199.8	1251.1	1422.3	0.68	0.90	1.04

**Table 10:** Reconstruction parameters.

Parameter	Value
Pixel Size	13.53217 µm
Smoothing	2 pix
Ring Artefact Correction	19
Beam Hardening Correction	41%
Minimum for CS to Image Conversion	0.000
Maximum for CS to Image Conversion	0.030

**Table 11:** Summary of analysis of micro-computed tomography images.

Parameter	Value in Sample:		
	“1”	“2”	“3”
Mean density [kg/m^3^]	510	519	619
Mean density of early wood [kg/m^3^]	410	367	463
Mean density of late wood [kg/m^3^]	912	814	934
Volume fraction of early wood [%]	80.1	66.2	66.9
Volume fraction of late wood [%]	19.8	33.6	33.1
Mean thickness of early growth [mm]	1.29	1.34	1.26
Mean thickness of late growth [mm]	0.35	0.70	0.56

**Table 12:** Selected results for different samples.

Testing Method	Parameter	Value in Sample:		
		“1”	“2”	“3”
Laboratory test	Mean density (at moisture content 18%) [kg/m^3^]	511.5	514.4	623.0
Acoustic method (Fakopp)	Mean MOE_dyn_ perpendicular to the grain [GPa]	0.98	0.77	1.6
Resistance drilling method	Mean RM [%]	16.5	16.3	16.9
	Mean FM [%]	47.4	47.7	55.3
